# Compulsive Hoarding Symptoms and the Role of Mindfulness Skills During Social Distancing for the COVID-19 Pandemic: An Exploratory Survey

**DOI:** 10.3389/fpsyt.2021.634454

**Published:** 2021-06-14

**Authors:** Donatella Marazziti, Andrea Pozza, Federico Mucci, Davide Dettore

**Affiliations:** ^1^Department of Clinical and Experimental Medicine, Section of Psychiatry, University of Pisa, Pisa, Italy; ^2^Saint Camillus International University of Health and Medical Sciences, Rome, Italy; ^3^BRF Foundation, Lucca, Italy; ^4^Department of Medical Sciences, Surgery and Neurosciences, University of Siena, Siena, Italy; ^5^Department of Biotechnology, Chemistry and Pharmacy, University of Siena, Siena, Italy; ^6^Department of Health Sciences, University of Florence, Florence, Italy

**Keywords:** COVID-19 pandemic, social distancing, coronavirus, compulsive hoarding, behavioral addiction, mindfulness, obsessive - compulsive disorder

## Abstract

People reporting compulsive hoarding symptoms (CHS) have lower mindfulness skills than those without such symptoms. Mindfulness skills can have the role of a protective buffer against stressful periods. The quarantine imposed to contain the COVID-19 spread had a negative impact on daily habits and healthy behaviors (including social interactions). An increased attachment to objects might be one of the under-recognized psychological consequences of these difficult times, yet no study focused on CHS. Through an online survey in men who were on quarantine during the pandemic, this exploratory survey examined the prevalence of men reporting CHS during this period and explored the role of mindfulness skills on CHS controlling for anxious-depressive/stress symptoms. Forty-three men from the general population completed the Obsessive Compulsive Inventory-Revised (OCI-R), Cognitive and Affective Mindfulness Scale-Revised (CAMS-R) and Depression Anxiety Stress Scales-21 (DASS-21). Twenty-eight percent reported CHS. No differences on the scores of the questionnaires emerged between men with and without CHS, except on CAMS-R Attention scores. In a logistic regression analysis lower CAMS-R Attention scores predicted CHS (β = −0.34, *p* = 0.03). This is the first, yet preliminary investigation on CHS during quarantine. The prevalence of CHS appears higher than the rates (4%) reported in the last years before the COVID-19 outbreak. Perhaps people showed more intense hoarding tendencies during quarantine/social distancing, and this pattern should be monitored. Larger samples, longitudinal designs and clinician-rated instruments are needed to support or not our findings.

## Introduction

During the last year, the outbreak of the COVID-19 pandemic has dramatically impacted on the societies of most countries worldwide (1). To cope with the spread of the infection, several national governments adopted a series of countermeasures including social distancing, more or less severe moving and activities restrictions, and quarantine. This social change represented and still represents a highly stressful life event with a negative impact on daily habits and healthy behaviors including social interactions. Therefore, it may potentially favor the onset of symptoms in individuals with a pre-existing vulnerability toward psychopathological conditions (2–6).

Once classified as a symptom dimension of obsessive–compulsive disorder (OCD) (7), hoarding disorder (HD) is now included as a separate psychopathological category in the OCD and Related Disorders chapter of DSM-5 (8). The clinical picture consists of a persistent and distressing difficulty discarding possessions, regardless of their actual value, due to a perceived need to save them. This behavioral pattern results in the accumulation of items that clutter living areas and compromises their intended use, causing significant impairment in social, occupational, or other areas of functioning. According to a recent review, around 2% of the general population meets the criteria for a full HD diagnosis, prevalence rates do not substantially change across developed countries and, it may increase with age (9). The prevalence of clinically significant compulsive hoarding symptoms (CHS) in people who do not meet the criteria for a full diagnosis was identified in 4–6% of the general population, and it was greater in older than younger age groups, greater in men than women (10).

The HD causes an important impairment in the quality-of-life levels of individuals (11), and it imposes a significant burden on their family members that is comparable with that experienced by natural caregivers of dementia people (12, 13). The HD is associated with high societal costs and its public health consequences include lack of hygiene and bad odors: it also contributes to the faster deterioration of buildings, infection of dwellings with rodents and insects and increased fire hazards (14–16). Like other obsessive-compulsive spectrum disorders, HD is often an under-recognized and untreated pathological condition (17). According to some studies [e.g., (18)], people suffering from HD may wait for a long time before attending a mental health facility or seeking professional help. In addition, most of them may be not enough aware of their symptoms due to social stigma and poor mental health literacy (19). Therefore, early identification of vulnerable cases seems to be a crucial public health strategy, particularly during a difficult period for healthcare services like the present one.

Mindfulness skills are a protective factor against stressful situations and periods that include the ability of staying in the present moment in a non-judgemental way (20). Being mindful means to be aware of both external and internal stimuli, and wittingly re-direct one's attention to the present moment, so that one is neither overwhelmed by the violence of thoughts, emotions, and sensations, nor led in one's actions and choices by those cognitive contents and affects. Several different definitions of mindfulness share one common element: the non-judgemental attitude toward one's inner experience (21, 22). Recent evidence showed the potentially protective role of mindfulness skills against the development and maintenance of psychological distress during the pandemic, but not only, in various populations [e.g., (23–25)]. Previous evidence suggested that CHS people show lower mindfulness skills, as compared with those not reporting such symptoms (26).

In conclusion, CHS represent a problematic, often under-recognized and under-reported, condition that significantly interferes with quality of life. Thus, there is a strong need for a better knowledge of the psychological factors which can protect from the development and maintenance of this condition during a difficult time like the present one.

### Rationale and Aims

The quarantine imposed by the governments to contain the COVID-19 spread represents a dramatic social change with a potentially severe impact on daily habits and healthy behaviors (including social interactions). An increased attachment to possessions and objects might be an under-recognized mental health negative outcome of these difficult times. Although there is a great effort to investigate the mental health effects of the quarantine, no study focused on CHS. In particular, it seems to be of great relevance to explore the psychological factors potentially related to a lower level of psychopathological conditions during the pandemic (27). A recent umbrella review suggested that, despite the quite large amount of data, more evidence is needed about the protective factors associated with OCD-related disorders or traits (28).

Based upon an online survey in a group of men who were in quarantine during the COVID-19 pandemic, the present exploratory survey examined the prevalence of men reporting clinically significant CHS during this particular period. In addition, the role of the mindfulness skills on the presence of clinically relevant CHS was explored.

## Methods

### Eligibility Criteria and Recruitment Procedure

Eligibility criteria included the fact that participants had provided written informed consent and declared to be in quarantine. Participation was anonymous, voluntary, and uncompensated. The data of this study represent a secondary analysis of a larger web-based online study which was conducted via Google form and aimed to explore the broad OCD-related features in the Italian general population during the COVID-19 pandemic. Participants were recruited through convenience sampling. Specifically, the web-based advertisement of the study was spread from 9th March 2020 to the end of April 2020, the period in which the complete quarantine was imposed by the Italian government. The advertisement was posted on a series of Facebook online groups, where the objectives, the target population, the characteristics of the self-report instruments and the fact that anonymity was assured were presented. All participants were in complete quarantine imposed by the national government to cope with the spread of the COVID-19.

Forty-three men recruited from the general population responded to an online survey about the quarantine mental health effects and completed a series of self-report questionnaires.

The study was approved by the Ethics Committee of the University where it was conducted.

### Measures

#### Obsessive Compulsive Inventory-Revised (OCI-R)

The OCI-R (29) measures the severity of obsessive-compulsive symptoms using 18 items grouped into six subscales assessing six subtypes (Washing, Obsessing, Hoarding, Ordering, Checking, and Mental Neutralizing) through a 5-point Likert scale (0 = Not at all, 4 = Extremely) (29). The Italian version showed acceptable to good internal consistency (Cronbach's alpha > 0.70 for all the subscales), and test-retest reliability (Pearson's *r* > 0.70) (30).

#### Cognitive and Affective Mindfulness Scale-Revised (CAMS-R)

It is a 12-item scale that measures everyday mindfulness and focuses on the degree to which respondents experience their thoughts and feelings (31). Items are rated on a 4-point Likert scale from 1 (rarely/not at all) to 4 (almost always). Scores on the scale are summed. Higher scores reflect greater mindfulness. Internal consistency across the 12 items was acceptable to good for two student samples (alpha = 0.74–0.80). The Italian version (32) showed four subscales with acceptable internal consistency including Attention (i.e., the ability to regulate attention), Present Focus (i.e., the orientation to present experience), Awareness (awareness of experience) and Acceptance (i.e., the attitude of acceptance or non-judgment toward experience).

#### Depression Anxiety Stress Scales-21 (DASS-21)

The DASS-21 (33) is a measure of psychological distress and comprises three subscales measuring depression, anxiety, and stress, respectively. All the scales comprise seven items each. Participants rated the extent to which the item applied to them over the last week on a 4-point Likert scale ranging from 0 (did not apply to me at all) to 3 (applied to me very much, or most of the time). The total scores for each scale are calculated by summing scores on the seven items and multiplying the total by two. The DASS-21 has very good psychometric properties (34). The Italian version showed good internal consistency (35).

### Data Analyses

Participants with clinically significant CHS were identified if they reported a score on the OCI-R Hoarding subscale higher than the 95th percentile of the normal distribution reported in the validation study (30). Group differences were tested by a series of one-way analyses of variance (ANOVAs), specifically the differences on age, the scores on the CAMS-R and DASS-21 between participants with and without clinically significant CHS. Cohen's *d* indices were calculated as effect sizes and they were interpreted according to the following criteria: values equal to 0.80 or higher were interpreted as large, values up to 0.50 as medium, and values up to 0.20 as small (36). Non-parametric tests were used to examine between-group differences on socio-demographics. Finally, a logistic regression analysis was carried out entering as predictors the scores on the CAMS-R and/or DASS-21 subscale scores that had a significant *p*-value in the ANOVAs and/or a large effect size, and the group categories (participants with and without clinically significant CHS) as outcome. The data analyses were conducted through the Statistical Packages for Social Sciences (SPSS) 25,00 version.

## Results

### Sociodemographic and Clinical Characteristics of the Group

Forty-three young men were included in the study (Table 1). Mean age was 25.77 years (SD = 4.40) ranging from 19 to 39. Twelve participants (27.9% of the group) reported clinically significant CHS, as shown by a score higher than the 95th percentile of the OCI-R Hoarding subscale scores of the community distribution reported in the validation paper of the measure (30).

**Table 1:** Demographic characteristics of participants (*n* = 43).

	***M* (SD; range)**	***n* (%)**
Age (years)	25.77 (4.40; 19–39)	
**Marital status**		
Single		21 (48.8)
Engaged or married		22 (51.2)
**Occupational status**		
Student		25 (58.1)
Working		16 (37.2)
Other		2 (4.7)
**Education level**		
Middle school		5 (11.6)
High school		24 (55.8)
Degree		5 (11.6)
**Compulsive hoarding symptoms**		
**(OCI-R Hoarding subscale score** **≥** **95th percentile of the normal distribution)**		
Yes		12 (27.9)
No		31 (72.1)

*M, mean; n, number of participants; OCI-R, obsessive compulsive inventory-revised; SD, standard deviation*.

### Group Differences and Effects of Mindfulness Skills on CHS

No differences were found between men with and without CHS on socio-demographic variables including age [*F*_(1, 41)_ = 0.88, *p* = 0.35], marital status [$\chi_{\left(1\right)}^{2}$ = 0.009, *p* = 0.92], occupational status [Kruskal-Wallis *H*_(1)_ = 0.61, *p* = 0.43], and education level [Kruskal-Wallis *H*_(1)_ = 1.52, *p* = 0.21].

Significant differences between men with and without CHS emerged only on the scores of the CAMS-R Attention with a large effect size, but not on the scores of the CAMS-R or DASS-21 (Table 2).

**Table 2:** Comparison between men with and without CHS on the clinical scales (*n* = 43).

	**Compulsive hoarding symptoms** ** (OCI-R Hoarding subscale score ≥ 95^**th**^ percentile of the normal distribution)**	**Mean**	**SD**	**95% CI**	***F_**(*df*)**_***	***p*-value**	**Cohen's *d***	
				**Lower**	**Upper**			
CAMS-R Attention	Yes (*n* = 31)	8.29	2.003	7.56	9.03	5.43_(1.41)_	0.025	−0.80
	No (*n* = 12)	6.50	2.844	4.69	8.31			
CAMS-R Present Focus	Yes (*n* = 31)	7.61	2.201	6.81	8.42	0.68_(1.41)_	0.41	−0.28
	No (*n* = 12)	7.00	2.132	5.65	8.35			
CAMS-R Acceptance	Yes (*n* = 31)	8.52	2.249	7.69	9.34	0.33_(1.41)_	0.56	−0.20
	No (*n* = 12)	8.08	2.021	6.80	9.37			
CAMS-R Awareness	Yes (*n* =31)	8.19	1.990	7.46	8.92	2.51_(1.41)_	0.12	−0.53
	No (*n* = 12)	7.17	1.642	6.12	8.21			
DASS-21 Depression	Yes (*n* = 31)	6.29	6.198	4.02	8.56	0.91_(1.41)_	0.34	0.32
	No (*n* = 12)	8.17	4.387	5.38	10.9			
DASS-21 Anxiety	Yes (*n* = 31)	3.29	3.514	2.00	4.58	1.42_(1.41)_	0.23	0.40
	No (*n* = 12)	4.67	3.025	2.74	6.59			
DASS-21 Stress	Yes (*n* = 31)	8.26	5.899	6.09	10.42	1.71_(1.41)_	0.19	0.44
	No (*n* = 12)	10.67	3.774	8.27	13.06			

*CAMS-R, cognitive and affective mindfulness scale-revised; CHS, compulsive hoarding symptoms; CI, confidence interval; d, effect size; DASS-21, depression anxiety stress scales-21 items; OCI-R, obsessive compulsive inventory-revised; SD, standard deviation*.

The logistic regression analysis included only the scores on the CAMS-R Attention which resulted associated with a significant *p*-value and a large effect size in the ANOVA. The results of this analysis showed that lower scores on the CAMS-R Attention scores predicted the presence of CHS (β = −0.34, Wald = 4.55, *p* = 0.03): individuals with lower CAMS-R Attention scores were more likely to have CHS.

## Discussion

The present exploratory study is the first empirical contribution investigating CHS in a group of men of the general population during the quarantine. The prevalence of such symptoms (28%) appears higher than the rates (4–6%) generally reported in the last years before the COVID-19 outbreak in the general population (10). This finding suggests that perhaps people have more intense hoarding behaviors during quarantine and social distancing, and this behavioral pattern should be more carefully monitored during the pandemic. As already reported (18), CHS are generally under-recognized by practitioners and under-reported by the individuals themselves. Such an increase of CHS during the quarantine might be attributed to a variety of factors including stocking of masks, soaps, sanitizers, disinfectants that can lead to CHS, increased stress subsequent to quarantine and nation-wide lockdown in response to the COVID-19, a lower chance for interpersonal contacts that increases people's attachment to objects, and a higher chance for compulsive online shopping as a way to cope with quarantine-related distress and loneliness (27).

The present preliminary findings suggest that the ability to regulate attention can protect from CHS and play the role of a psychological factor associated with a lower level of CHS during this dramatic social change when the individual may not interact with people and must stay at home. This potentially protective role of the attention facet of mindfulness skills appears consistent with an increasing amount of data which show the relation between a higher level of this mindfulness skill and a lower level of psychological distress during the pandemic in various populations [e.g., (24, 25)]. It might be speculated that an attitude based upon attention regulation can be associated with an increased distress tolerance and regulation which has been found to be a significant predictor of CHS (37–39). However, the other mindfulness skills were not predictive of CHS, specifically the orientation to present or immediate experience, the awareness of experience, and an attitude of acceptance or non-judgment toward experience. In contrast with previous data (40, 41), we did not detect any differences on anxious-depressive symptoms and stress levels on CHS that prevented the inclusion of these features as predictors in the regression analysis. However, not all the previous studies confirmed that distress levels are higher amongst people with HD or CHS. For example, Worden et al. (42) found that distress levels did not discriminate a clinical group with HD from a control group after controlling for depressive and anxious symptoms. One possible explanation for this result is that the CHS group was not composed of individuals who sought help for CHS; for this reason, maybe the level of distress in this group was not high. An alternative explanation might be that both the groups were in quarantine when they completed the survey, and they were not compared on distress levels with another group who was not in quarantine. As observed elsewhere, the quarantine may increase the likelihood that people with obsessive-compulsive spectrum conditions develop psychological distress (43).

Since the present one was an exploratory survey, some important limitations should be pointed out.

Firstly, the small sample size prevented the assessment of the effects of further variables. For example, it might be interesting to investigate the effects of other variables related to CHS, such as attachment styles, or other psychopathological symptoms potentially overlapped with CHS such as Internet addiction symptoms, compulsive shopping symptoms and obsessive-compulsive symptoms (44–46). Another key aspect to be noted is that if we used a Bonferroni correction to test the ANOVA-based comparisons, Bonferroni-adjusted *p*-value would be 0.007 (=0.05/7), thus the observed significance for CAMS-R Attention (*p* = 0.025) would be lost. The small sample size might be a cause of this problem. In addition, perhaps the lack of significant effects of some predictors in the logistic regression analysis might be attributed to the low power of the statistical analysis. Therefore, future research should include larger samples. The cross-sectional design did not allow a causal relationship to be established. Therefore, it may be interesting to explore whether specific mindfulness skills can predict the onset of CHS over time in prospective studies during the pandemic. Moreover, by using a longitudinal design it would be important to understand whether, or not the quarantine can increase the risk of developing CHS. For example, it would be interesting to explore whether the reduction of social contacts during the quarantine and social distancing might be a mediator of an increased risk of CHS, since social relationships and support have a protective effect against obsessive-compulsive spectrum symptoms (47–49).

Another relevant shortcoming regards the use of self-report measures. Future research should integrate self-report instruments with clinician-administered tools (e.g., interviews). In addition, despite CHS are more likely to be present among men, future research should include also a group of women and explore the potential role of gender.

In conclusion, this is the first investigation on CHS during quarantine. The prevalence of CHS appears higher than the rates reported in the last years before the COVID-19 outbreak. Perhaps people have more intense hoarding tendencies during quarantine/social distancing, and this pattern should be monitored further. Larger samples, longitudinal designs and clinician-rated instruments are needed to support or not our findings.

## Data Availability Statement

The raw data supporting the conclusions of this article will be made available by the authors, without undue reservation.

## Ethics Statement

The studies involving human participants were reviewed and approved by University of Florence. The patients/participants provided their written informed consent to participate in this study.

## Author Contributions

DM designed the study, conducted the literature searches, collected and analyzed the data, wrote the first draft of the paper, and edited the final version. AP designed the study, conducted the literature searches, collected and analyzed the data, and wrote the first draft of the paper. FM designed the study, analyzed the data, and edited the final version of the paper. DD designed the study, collected the data, wrote the first draft, and edited the final version of the paper. All authors contributed to the article and approved the submitted version.

## Conflict of Interest

The authors declare that the research was conducted in the absence of any commercial or financial relationships that could be construed as a potential conflict of interest.

## References

[1] Giorli A Ferretti F Biagini C Salerni L Bindi I Dasgupta S. A literature systematic review with meta-analysis of symptoms prevalence in Covid-19: the relevance of olfactory symptoms in infection not requiring hospitalization. Curr Treat Opt Neurol. (2020) 22:1–14. 10.1007/s11940-020-00641-5 PMC7453082 32874091

[2] Greenberg N Docherty M Gnanapragasam S Wessely S. Managing mental health challenges faced by healthcare workers during covid-19 pandemic. BMJ. (2020) 368:m1211. 10.1136/bmj.m1211 32217624

[3] Marazziti D Mucci F Piccinni A Dèttore D Pozza A. Covid-19 outbreak: a challenge calling for early intervention on contamination obsessive fears? BPA Appl Psychol Bull. (2020) 67:62–70. 10.26387/bpa.285.6

[4] Marazziti D Pozza A Di Giuseppe M Conversano C. The psychosocial impact of COVID-19 pandemic in Italy: a lesson for mental health prevention in the first severely hit European country. Psychol Trauma Theory Res Pract Pol. (2020) 12:531–3. 10.1037/tra0000687 32525387

[5] Pierce M Hope H Ford T Hatch S Hotopf M John A. Mental health before and during the COVID-19 pandemic: a longitudinal probability sample survey of the UK population. Lancet Psychiatry. (2020) 7:883–92. 10.1016/S2215-0366(20)30308-4 32707037 PMC7373389

[6] Pozza A Mucci F Marazziti D. Risk for pathological contamination fears at coronavirus time: proposal of early intervention and prevention strategies. Clin Neuropsychiatry. (2020) 17:100–2. 10.36131/CN20200214 PMC8629035 34908978

[7] Pozza A Barcaccia B Dèttore D. The Obsessive Compulsive Inventory-Child Version (OCI-CV): further evidence on confirmatory factor analytic structure, incremental and criterion validity in Italian community children and adolescents. Arch Psychiatr Nurs. (2017) 31:291–5. 10.1016/j.apnu.2017.02.003 28499570

[8] American Psychiatric Association. Diagnostic and Statistical Manual of Mental Disorders. 5th ed. Washington, DC: American Psychiatric Association (2013).

[9] Postlethwaite A Kellett S Mataix-Cols D. Prevalence of hoarding disorder: a systematic review and meta-analysis. J Affect Disord. (2019) 256:309–16. 10.1016/j.jad.2019.06.004 31200169

[10] Bulli F Melli G Carraresi C Stopani E Pertusa A Frost RO. Hoarding behaviour in an Italian non-clinical sample. Behav Cogn Psychother. (2014) 42:297–311. 10.1017/S1352465812001105 23286647

[11] Coluccia A Fagiolini A Ferretti F Pozza A Goracci A. Obsessive-Compulsive Disorder and quality of life outcomes: protocol for a systematic review and meta-analysis of cross-sectional case-control studies. Epidemiol Biostat Public Health. (2015) 12:e11037–1. 10.2427/10037

[12] Drury H Ajmi S de la Cruz LF Nordsletten AE Mataix-Cols D. Caregiver burden, family accommodation, health, and well-being in relatives of individuals with hoarding disorder. J Affect Disord. (2014) 159:7–14. 10.1016/j.jad.2014.01.023 24679383

[13] Subramaniam M Abdin E Vaingankar JA Picco L Chong SA. Hoarding in an Asian population: prevalence, correlates, disability and quality of life. Ann Acad Med. (2014) 43:535–43. 25523857

[14] Chapin RK Sergeant JF Landry ST Koenig T Leiste M Reynolds K. Hoarding cases involving older adults: the transition from a private matter to the public sector. J Gerontol Soc Work. (2010) 53:723–42. 10.1080/01634372.2010.517697 20972928

[15] Frost RO Steketee G Williams L. Hoarding: a community health problem. Health Soc Care Commun. (2000) 8:229–34. 10.1046/j.1365-2524.2000.00245.x 11560692

[16] McGuire JF Kaercher L Park JM Storch EA. Hoarding in the community: a code enforcement and social service perspective. J Soc Serv Res. (2013) 39:335–44. 10.1080/01488376.2013.770813

[17] Pozza A Albert U Dèttore D. Perfectionism and intolerance of uncertainty are predictors of OCD symptoms in children and early adolescents: a prospective, cohort, one-year, follow-up study. Clin Neuropsychiatry. (2019) 16:53–61. PMC8650182 34908939

[18] Bodryzlova Y O'Connor K. Factors affecting the referral rate of the hoarding disorder at primary mental health care in Quebec. Commun Mental Health J. (2018) 54:773–81. 10.1007/s10597-018-0234-z 29353402

[19] Bates S De Leonardis AJ Corrigan PW Chasson GS. Buried in stigma: experimental investigation of the impact of hoarding depictions in reality television on public perception. J Obsessive Comp Relat Disord. (2020) 26:100538. 10.1016/j.jocrd.2020.100538

[20] Baer RA. Mindfulness training as a clinical intervention: conceptual and empirical review. Clin Psychol Sci Pract. (2003) 10:125–43. 10.1093/clipsy.bpg015

[21] Barcaccia B Baiocco R Pozza A Pallini S Mancini F Salvati M. The more you judge the worse you feel. A judgemental attitude towards one's inner experience predicts depression and anxiety. Person Ind Differ. (2019) 138:33–9. 10.1016/j.paid.2018.09.012

[22] Jennings JL Apsche JA. The evolution of a fundamentally mindfulness-based treatment methodology: from DBT and ACT to MDT and beyond. Int J Behav Consult Therapy. (2014) 9:1–3. 10.1037/h0100990

[23] Conversano C Orrù G Pozza A Miccoli M Ciacchini R Marchi L. Is mindfulness-based stress reduction effective for people with hypertension? A systematic review and meta-analysis of 30 years of evidence. Int J Environ Res Public Health. (2021) 18:2882. 10.3390/ijerph18062882 33799828 PMC8000213

[24] Hong W Liu RD Ding Y Fu X Zhen R Sheng X. Social media exposure and college students' mental health during the outbreak of CoViD-19: the mediating role of rumination and the moderating role of mindfulness. Cyberpsychol Behav Soc Netw. (2020) 24:282–7. 10.1089/cyber.2020.0387 33050721

[25] Pálvölgyi Á Makai A Prémusz V Trpkovici M Ács P Betlehem J. A preliminary study on the effect of the COVID-19 pandemic on sporting behavior, mindfulness and well-being. Health Prob Civil. (2020) 14:157–64. 10.5114/hpc.2020.97898

[26] Ong CW Krafft J Levin ME Twohig MP. An examination of the role of psychological inflexibility in hoarding using multiple mediator models. J Cogn Psychother. (2018) 32:97–111. 10.1891/0889-8391.32.2.97 32746400

[27] Banerjee D. The other side of COVID-19: impact on obsessive compulsive disorder (OCD) and hoarding. Psychiatry Res. (2020) 288:112966. 10.1016/j.psychres.2020.112966 32334276 PMC7151248

[28] Fullana MA Tortella-Feliu M de la Cruz LF Chamorro J Pérez-Vigil A Ioannidis JP. Risk and protective factors for anxiety and obsessive-compulsive disorders: an umbrella review of systematic reviews and meta-analyses. Psychol Med. (2020) 50:1300–15. 10.1017/S0033291719001247 31172897

[29] Foa EB Huppert JD Leiberg S Langner R Kichic R Hajcak G. The obsessive-compulsive inventory: development and validation of a short version. Psychol Assess. (2002) 14:485–96. 10.1037/1040-3590.14.4.485 12501574

[30] Marchetti I Rocco Chiri L Ghisi M Sica C. Obsessive–Compulsive Inventory–Revised (OCI-R): presentazione e indicazione di utilizzo nel contesto italiano. Psicoterapia Cogn Comp. (2010) 16:69–84.

[31] Feldman G Hayes A Kumar S Greeson J Laurenceau JP. Mindfulness and emotion regulation: the development and initial validation of the Cognitive and Affective Mindfulness Scale-Revised (CAMS-R). J Psychopathol Behav Assess. (2007) 29:177–90. 10.1007/s10862-006-9035-8

[32] Veneziani CA Voci A. The Italian adaptation of the cognitive and affective mindfulness scale-revised. Test Psychometr Methodol Appl Psychol. (2015) 22:43–52. 10.4473/TPM22.1.4

[33] Lovibond SH Lovibond PF. Manual for the Depression Anxiety Stress Scales. 2nd ed. Sydney, NSW: Psychology Foundation of Australia (1995).

[34] Henry JD Crawford JR. The short-form version of the Depression Anxiety Stress Scales (DASS-21): construct validity and normative data in a large non-clinical sample. Br J Clin Psychol. (2005) 44:227–39. 10.1348/014466505X29657 16004657

[35] Bottesi G Ghisi M Altoè G Conforti E Melli G Sica C. The Italian version of the Depression Anxiety Stress Scales-21: factor structure and psychometric properties on community and clinical samples. Compr Psychiatry. (2015) 60:170–81. 10.1016/j.comppsych.2015.04.005 25933937

[36] Cohen J. Statistical Power Analysis for the Behavioral Sciences. New York, NY: Routledge (1988).

[37] Grisham JR Roberts L Cerea S Isemann S Svehla J Norberg MM. The role of distress tolerance, anxiety sensitivity, and intolerance of uncertainty in predicting hoarding symptoms in a clinical sample. Psychiatry Res. (2018) 267:94–101. 10.1016/j.psychres.2018.05.084 29886277

[38] Timpano KR Buckner JD Richey JA Murphy DL Schmidt NB. Exploration of anxiety sensitivity and distress tolerance as vulnerability factors for hoarding behaviors. Depress Anxiety. (2009) 26:343–53. 10.1002/da.20469 19123454 PMC2770430

[39] Tolin DF Levy HC Wootton BM Hallion LS Stevens MC. Hoarding disorder and difficulties in emotion regulation. J Obsess Comp Relat Disord. (2018) 16:98–103. 10.1016/j.jocrd.2018.01.006 30828541 PMC6391883

[40] Phillips KA Stein DJ Rauch SL Hollander E Fallon BA Barsky A. Should an obsessive–compulsive spectrum grouping of disorders be included in DSM-V? Depress Anxiety. (2010) 27:528–55. 10.1002/da.20705 20533367 PMC3985410

[41] Raines AM Short NA Fuller KL Allan NP Oglesby ME Schmidt NB. Hoarding and depression: the mediating role of perceived burdensomeness. J Psychiatr Res. (2016) 83:24–8. 10.1016/j.jpsychires.2016.08.003 27543825

[42] Worden B Levy HC Das A Katz BW Stevens M Tolin DF. Perceived emotion regulation and emotional distress tolerance in patients with hoarding disorder. J Obsess Comp Relat Disord. (2019) 22:100441. 10.1016/j.jocrd.2019.100441 32818134 PMC7430655

[43] Prestia D Pozza A Olcese M Escelsior A Dèttore D Amore M. The impact of the COVID-19 pandemic on patients with OCD: effects of contamination symptoms and remission state before the quarantine in a preliminary naturalistic study. Psychiatry Res. (2020) 291:113213. 10.1016/j.psychres.2020.113213 32535508 PMC7280119

[44] Boerema YE de Boer MM van Balkom AJ Eikelenboom M Visser HA van Oppen P. Obsessive compulsive disorder with and without hoarding symptoms: characterizing differences. J Affect Disord. (2019) 246:652–8. 10.1016/j.jad.2018.12.115 30611063

[45] Mathes BM Timpano KR Raines AM Schmidt NB. Attachment theory and hoarding disorder: a review and theoretical integration. Behav Res Ther. (2020) 125:103549. 10.1016/j.brat.2019.103549 31923776

[46] Pozza A Coluccia A Kato T Gaetani M Ferretti F. The ‘Hikikomori' syndrome: worldwide prevalence and co-occurring major psychiatric disorders: a systematic review and meta-analysis protocol. BMJ Open. (2019) 9:e025213. 10.1136/bmjopen-2018-025213 31542731 PMC6756420

[47] Medard E Kellett S. The role of adult attachment and social support in hoarding disorder. Behav Cogn Psychother. (2014) 42:629–33. 10.1017/S1352465813000659 24103104

[48] Zimmermann M Chong AK Vechiu C Papa A. Modifiable risk and protective factors for anxiety disorders among adults: a systematic review. Psychiatry Res. (2020) 285:112705. 10.1016/j.psychres.2019.112705 31839417

[49] Pozza A Dettore D. Drop-out and efficacy of group versus individual cognitive behavioural therapy: what works best for obsessive-compulsive disorder? A systematic review and meta-analysis of direct comparisons. Psychiatry Res. (2017) 258:24–36. 10.1016/j.psychres.2017.09.056 28982038

